# The Effects of Using a Nature-Sound Mobile Application on Psychological Well-Being and Cognitive Performance Among University Students

**DOI:** 10.3389/fpsyg.2021.699908

**Published:** 2021-11-24

**Authors:** Jiutong Luo, Minhong Wang, Ling Chen

**Affiliations:** ^1^Advanced Innovation Center for Future Education, Faculty of Education, Beijing Normal University, Beijing, China; ^2^Center for Educational Science and Technology, Beijing Normal University at Zhuhai, Zhuhai, China; ^3^Health Science Center, Shenzhen University, Shenzhen, China; ^4^KM&EL Lab, Faculty of Education, The University of Hong Kong, Pokfulam, Hong Kong SAR, China; ^5^Department of Educational Information Technology, East China Normal University, Shanghai, China; ^6^Faculty of Education, School of Educational Technology, Beijing Normal University, Beijing, China

**Keywords:** nature sounds, mobile application, psychological well-being, cognitive performance, university students

## Abstract

Many university students have been struggling with multiple challenges that may cause mental fatigue. Exposure to the natural environment is found to have restorative effects on mental fatigue, which can be explained by its benefits in physiological, psychological, and cognitive aspects. While the natural environment contains both visual and auditory elements, research on the effects of auditory elements, such as nature sounds, is underdeveloped and limited to laboratory settings. It remains unclear what are the effects of exposure to nature sounds in daily life settings. The study was conducted with 71 students from a university, who were randomly assigned to the experimental group using a nature-sound mobile application in daily life and the control group not using the application. After a 4-week exposure to the intervention, the students in the experimental group outperformed their counterparts in the control group on psychological well-being reflected in positive affect, as well as cognitive performance reflected in flow state, attention (in terms of alerting) and working memory (in terms of accuracy and reaction time). The findings reveal the positive impact of exposure to relaxing nature sounds on university students’ psychological well-being and cognitive performance, as well as the potential of mobile applications to provide easy exposure to nature sounds.

## Introduction

Modern society offers various opportunities for university students to learn, play, and live. Meanwhile, it has brought challenges to students in multiple aspects. *First*, university students are often busy with daily schedules such as attending lectures, working on projects, and participating in academic and social events (Abbott, 2015). They also experience external pressure in peer competition and job-seeking practices (Stieger et al., 2020). These experiences may make them feel mentally exhausted, so called mental fatigue (Kaplan, 1987) – the psychobiological state caused by prolonged periods of demanding cognitive activity (Marcora et al., 2009). *Second*, university students are expected to engage in effective learning for developing advanced knowledge and skills to solve real-world complex problems. Effective learning nowadays requires the processing of a large amount of information from multiple sources, which may place high-cognitive demand on learners (Sweller, 1994; van Merriënboer and Sweller, 2005). *Third*, the wide adoption of information and communication technologies (ICTs) has significantly changed the daily life and entrainment experiences of human beings including university students (Lips et al., 2017; Lu et al., 2019). Engagement in the extensive use of ICTs or media multitasking is likely to exert a negative influence on students’ psychological well-being and cognitive performance (e.g., Young, 1998; Park et al., 2011; Shapiro and Margolin, 2014; Uncapher and Wagner, 2018; Luo et al., 2020a, 2020b).

In summary, mental fatigue could happen unconsciously along with university students’ daily lives. On the one hand, mental fatigue may lead to emotional dysregulation (Grillon et al., 2015; Manning et al., 2019), which has negative impact on psychological well-being. On the other hand, mental fatigue may affect the ability to maintain optimal cognitive performance, for example, the loss of attentional focus, lack of self-control, and poor decision-making performance (Kaplan, 1987; Abbott, 2015). How, then, might students manage mental fatigue? According to the literature, exposure to the natural environment, which contains both visual and auditory elements, has long been suggested by environmental psychologists as an approach to handling mental fatigue (Ulrich, 1983; Kaplan and Kaplan, 1989; Ulrich et al., 1991; Kaplan, 1995). With respect to auditory elements of the natural environment, the previous studies have shown promising effects of nature sounds in terms of perceived restorativeness (e.g., Payne, 2013; Ratcliffe et al., 2016) and cognitive performance (e.g., Abbott, 2015; Van Hedger et al., 2019; Barton, 2020). However, these studies focused on the effects of exposure to nature sounds in experimental environments. It remains unclear whether and to what extent exposure to nature sounds will affect psychological well-being and cognitive performance in daily life settings beyond laboratories. To this end, this study aimed to investigate the effects of a nature-sound mobile application on psychological well-being and cognitive performance among university students.

### The Benefits of Exposure to Natural Environments

The positive effect of exposure to nature has been widely reported (for reviews, see Abraham et al., 2010; Bowler et al., 2010; Kondo et al., 2018). According to the literature, there are two theories frequently referred to when investigating the benefits of exposure to nature – Stress Recovery Theory (SRT; Ulrich, 1983; Ulrich et al., 1991) and Attention Restoration Theory (ART; R. Kaplan and Kaplan, 1989; Kaplan, 1995). The first theory, i.e., SRT, derived from the psycho-evolutionary theory (Ulrich, 1983), suggests that cognitive fatigue may be a result of the stress in sustaining attention to tasks (Cohen and Spacapan, 1978). It predicts that natural environments have restorative effects in psychological (e.g., emotion) and physiological aspects (Ulrich et al., 1991). The second theory, i.e., RT, emphasizes the role of directed attention in human information processing (Kaplan and Kaplan, 1989; Kaplan, 1995). According to ART, exposure to natural environments that are characteristic of “being away,” “fascination,” “extent,” and “compatibility” can help to restore attention (Kaplan, 1995) and improve performances on cognitive tasks (Berman et al., 2008; Barton, 2020).

The two theories have explored the benefits of exposure to nature from psychological and cognitive perspectives and have been verified by empirical studies. In terms of the cognitive aspects, Berto (2005) demonstrated that viewing the photos of restorative environments, relative to nonrestorative environments or geometrical patterns, helped to maintain and restore the participants’ directed attention, or deliberately ignore distractions in the environment. Subsequent research has also found that presenting nature scenes (e.g., Hawaiian beach) through virtual reality to workers in open office settings helped them foster flow state (Ruvimova et al., 2020). Flow state, a positive experiential and mental state highly related to cognitive performance, occurs when the people are totally immersed in their activities (Csikszentmihalyi, 1990). In terms of psychological well-being, Hamann and Ivtzan (2016) reported that exposure to a natural environment for 30min per day for a period of 1month could improve positive affect (e.g., happiness, meaning in life, and mindfulness) and decrease negative affect (e.g., distress and upset).

### The Potential Effects of Nature Sounds

The natural environment has both visual and auditory elements, and the benefits of exposure to nature’s visual elements (e.g., natural scenery pictures) have been frequently demonstrated (Berto, 2005; Song et al., 2018). For example, Lee et al. (2015) reported that a short (i.e., a 40-s) view of a picture of flowering meadow with a green roof could be sufficient to boost sustained attention. Meanwhile, research on restorative effects of natural environments has been expanded from visual elements to auditory elements, by investigating the effects of nature sounds (Björk et al., 2008; Payne, 2013; Ratcliffe et al., 2013, 2016) such as the sounds of birds, rainfall, and waves. These sounds are the attachment of nature places and may exert a feeling of being in nature (e.g., Franco et al., 2017). According to the SRT and ART above (Ulrich, 1983; Kaplan and Kaplan, 1989; Ulrich et al., 1991; Kaplan, 1995), the nature sounds have been suggested to have similar benefits on psychological well-being and cognitive performance as the nature environment and nature scenery.

In this case, nature sounds, which only include the acoustic properties of the natural environment, are perceived to be pleasant and relaxing and have a link with perceived restoration (Ratcliffe et al., 2013, 2016, 2020; Shu and Ma, 2018). Largo-Wight et al. (2016) reported the benefits of a 7-min nature-sound intervention in psychological (e.g., self-reported stress) and physiological aspects (e.g., muscle tension and pulse rate). With respect to the cognitive aspects, it was found that nature sounds may help people to recall positive affect and memories (Barton, 2020) and improve working memory (Abbott, 2015). Van Hedger et al. (2019) also reported that the participants exposed to nature sounds outperformed those receiving urban sounds in terms of cognitive performance. In addition, nature sounds could induce brain connectivity changes and increase parasympathetic activity, which might relate to stress reduction (van Praag et al., 2017).

In general, research on the effects of nature-sound is underdeveloped. Existing studies have been mostly conducted in laboratory settings (Abbott, 2015; Largo-Wight et al., 2016; Van Hedger et al., 2019). It remains unclear whether exposure to nature sounds in a daily routine can improve students’ psychological well-being and cognitive performance.

### The Present Study

As discussed above, many university students have been struggling with multiple challenges that may cause mental fatigue. Exposure to the natural environment containing both visual and auditory elements has been suggested to have restorative effects on mental fatigue, which can be explained by its effects on physiological, psychological, and cognitive aspects. With respect to auditory elements such as nature sounds, research on the effects of nature-sound is underdeveloped and limited to laboratory settings. It remains unclear what are the effects of exposure to nature sounds in daily routine.

This study aimed to address the gap by investigating whether exposure to relaxing nature sounds in daily life has an impact on students. According to the above literature review, this study mainly focused on two aspects, i.e., psychological well-being and cognitive performance. The psychological well-being refers to positive and negative affect; while the cognitive performance includes flow state, attention, and working memory. In line with the theories (Ulrich, 1983; Kaplan and Kaplan, 1989; Ulrich et al., 1991; Kaplan, 1995) and empirical studies in laboratory settings (e.g., Abbott, 2015; Hamann and Ivtzan, 2016; Van Hedger et al., 2019; Ruvimova et al., 2020), this study hypothesized that the nature sounds could have restorative effects on students’ psychological well-being and cognitive performance. Therefore, this study used the pre- and post-test experimental design and collected the quantitative data. The study was conducted with university students, who were randomly assigned to the experimental group with exposure to relaxing nature sounds in their daily life through a free mobile application and the control group without such exposure for a period of 4 consecutive weeks. The effects of the intervention were investigated by assessing students’ psychological well-being (i.e., positive and negative affect) and cognitive performance (reflected in flow state, attention performance, and working memory performance).

This study focused on the followings research questions (RQs):

RQ1: Does exposure to nature sounds through a mobile application in daily life impact on psychological well-being among university students?
RQ2: Does exposure to nature sounds through a mobile application in daily life impact on cognitive performance among university students?
RQ2 includes the following sub-questions.
RQ2.1: Does exposure to nature sounds through a mobile application in daily life increase flow state among university students?
RQ2.2: Does exposure to nature sounds through a mobile application in daily life improve students’ attention performance?
RQ2.3: Does exposure to nature sounds through a mobile application in daily life improve students’ working memory performance?

## Materials and Methods

### Participants

As very few studies have explored the effects of nature sounds in daily life settings, this study aimed to estimate a conservative anticipated effect size of *ƒ*=0.20 after consulting experts in quantitative studies since the medium effect size (Cohen’s *ƒ*) would be around 0.20 (Cohen, 1992). The prior power calculations revealed that it required at least 52 participants to produce this effect size with a statistical power of 0.80, alpha of 0.05 (two-tailed), and correlation of 0.5 among repeated measures.

In this study, we recruited undergraduate students from a university in southeast China. In doing so, we sent an advertisement to all students in the university through social media and received responses from 76 students who expressed to have interest in participating in the study and reported to have normal hearing and normal or corrected to normal vision required for the study. The 76 students were randomly assigned to the experimental and control groups. In doing so, we used the SPSS software to give serial numbers (from 1 to 76) to all participants; we then used the “random sample of cases” function in the software to select approximately 50% of participants from the list. The selected participants were assigned to the experimental group and the rest were assigned to the control group. Five students did not complete the post-test, and their data were not used for analysis. In other words, 36 students in the experimental group and 35 others in the control group completed the study. The 71 participants included 28 males and 43 females; they were aged between 17 and 23years (mean=19.15, SD=1.24). The Chi-square test revealed that there were no gender differences between groups (*χ*^2^=0.15, *p*=0.70). This research was approved by the Ethics Review Committee of the researcher’s university. The participants provided their written informed consent to participate in this study. The students’ participation in this study was not related to their study program at the university. Students were allowed to withdraw from the study at any time without any consequences. Each participant received RMB80 after completing the study.

### Measures and Instruments

To investigate the effects of exposure to nature sounds, students’ psychological well-being, and cognitive performance were assessed before and after the study and compared between the experimental and control groups.

#### Psychological Well-Being

The participants’ psychological well-being was assessed by adopting the positive and negative affect schedule (PANAS; Watson et al., 1988). The PANAS includes 20 items measuring affective experience (i.e., perceived feelings and emotions) over the past 1–2weeks. Example descriptions of the positive affect are “*interested*,” “*excited*,” and “*proud*,” and example descriptions of the negative affect are “*distressed*,” “*guilty*,” and “*upset*.” They were all measured on a five-point scale (1=*Very slightly or not at all*; 5=*Extremely*). The Chinese version of this scale was validated in previous study (Huang et al., 2003). The reliability (Cronbach’s *α*) of the positive affect dimension in pre- and post-tests are 0.77 and 0.85, respectively. The reliability (Cronbach’s *α*) of the negative affect dimension in pre- and post-tests are 0.80 and 0.81, respectively.

#### Cognitive Performance

The participants’ cognitive performance was assessed through a survey on self-reported flow state and cognitive tests on attention and working memory performance. The cognitive tests were computerized with E-Prime 3.0 and performed individually by the participants on a 15-inch laptop. Students were asked to consider both time and accuracy when performing the tests.

##### Flow State

The participants’ flow state was measured by eight items adopted from the flow short scale (Rheinberg et al., 2003; Engeser and Rheinberg, 2008). This scale was developed and validated to measure self-reported flow state in general; and its Chinese version has been widely used (e.g., Bian et al., 2018). Example items of this scale are: “*I feel just the right amount of challenge*,” and “*I am totally absorbed in what I am doing*.” Students were asked to respond on a five-point scale (1=*Strongly disagree*; 5=*Strongly agree*) to report their overall tendency to experience flow when performing the cognitive tests. The reliabilities (Cronbach’s *α*) of this scale in pre- and post-tests are 0.80 and 0.75, respectively.

##### Attention Performance

**Figure 1:**
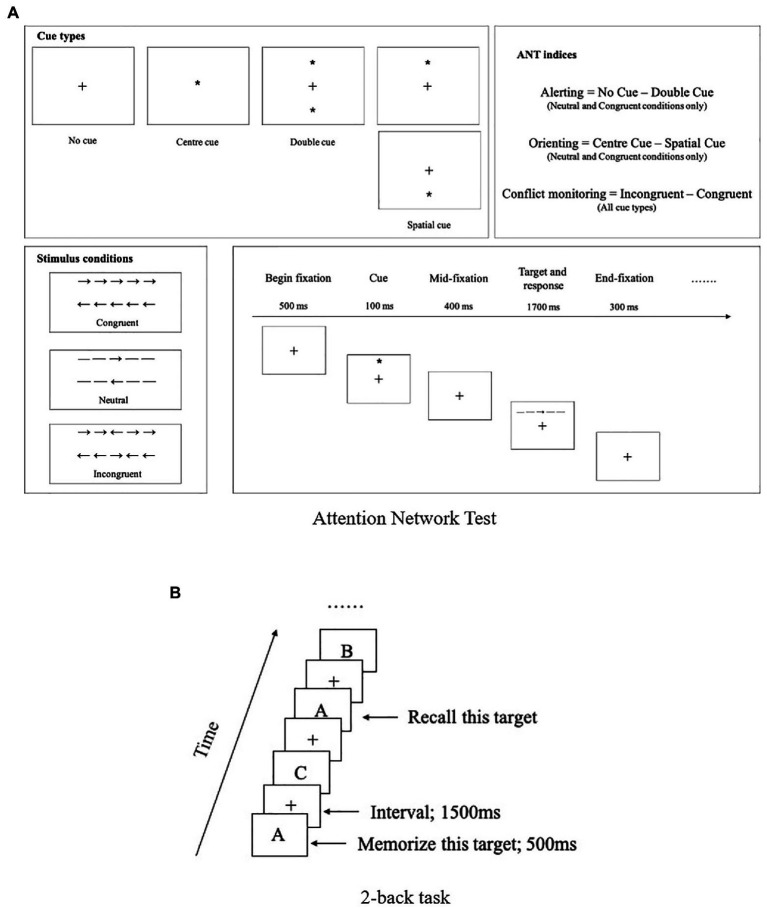
Experiment design for attention network test **(A)** and 2-back task **(B)**.

The Attention Network Test (ANT; Fan et al., 2002) was adopted to measure the participants’ attention performance. During the test, the participant was required to determine whether a central arrow on the screen points left or right (see Figure 1A). The participant’s performance could be influenced by alerting cues, spatial cues, and flankers displayed on the screen. Accordingly, the performance was measured in three indicators: alerting (the ability to achieve and maintain an alert state), orienting (the ability to select information from sensory input), and conflict monitoring (the ability to resolve conflict among responses).

The test included two blocks with each block containing 48 trials. The implementation of the test followed prior studies (Fan et al., 2002; Johnson et al., 2008) with modification in the following parameters: in each trial, the pre-fixation duration was set to 500ms, cue duration was set to 100ms, the mid-fixation duration was set to 400ms, and the target and response duration was set up to 1700ms, with an end-fixation duration of 300ms, as shown in Figure 1A.

##### Working Memory Performance

The cognitive test of working memory performance adopted a 2-back task including three blocks, with each block containing 30 trials. There are 12±1 targets across different blocks (average 40%). It is a task at an appropriate level of difficulty and is commonly used to measure working memory ability in cognitive studies among adults and adolescents (Wiradhany and Nieuwenstein, 2017; Luo et al., 2021). In the test, the participants were asked to respond regarding whether the currently displayed letter was the same as the second letter before. In this study, each letter was displayed for 500ms in the center of the screen, and then left blank with a cross (‘+’) in the center of the screen for 1,500ms (see Figure 1B). The participants were asked to respond by pressing a number button (1=“*Yes*,” 2=“*No*”). Their performance was measured in terms of accuracy (%) and reaction time (ms). The reaction time refers to the average time from each stimuli onset to the participant pressing the answer button.

### Procedure

The pre- and post-test experimental design was adopted for this study. At the beginning of the study, each of the students in the experimental and control groups was asked to complete a paper-based questionnaire containing personal information, psychological well-being (i.e., positive and negative affect), and flow state. After that, each student was required to perform two cognitive tests using a laptop, including the attention task and the working memory task. Task order was counterbalanced across participants to minimize order effects.

After the pre-test, students in the experimental group were introduced to a nature-sound mobile application. As mentioned, university students are often busy with many daily schedules, such as attending lectures, working on projects, and participating in academic and social events. Therefore, the participants in this study were asked to listen to the nature sounds for 30 consecutive minutes (i.e., one session) every day when they were working on academic related tasks for a period of 4weeks. The decision for setting a period of 4weeks was mainly due to the context of the study; that is, this study aimed to explore the effects of nature sounds among university students in their daily lives. It was thus not appropriate to set the period to be one session only in laboratory settings as other related studies. In this study, the period was set to 4weeks based on the common structure of academic terms. Usually, there is about 1-month time between the midterm exam and final exam, and it is appropriate to recruit students to participant in the study in this period.

The mobile application used in this study offers free open access to nature sounds. It contains computer simulated nature sounds in a number of categories including birds, rainfall, waves, and wind among others. Each sound piece can be automatically repeated with no time limits. The participant could choose one piece of nature sound to play, and they could play a sound piece as long as they liked although they were required to use the application for 30min only every time. They were requested to submit the screenshot of their mobile application records (including how much time and how many sessions they had used) on a daily basis. The participants reported that they often played this nature-sound application with headphones in study rooms where they need to keep quiet.

Meanwhile, students in the control group received no intervention during the whole procedure. However, they were introduced the same nature-sound mobile application after finishing the post-test. In this study, students in both groups had to spend a considerable amount of time to complete their academic tasks in a semester. The main difference between the two groups was whether to listen to the nature sounds for 30min per day when working on academic tasks during the 4-week study.

The fidelity of the study was measured by the amount of time and the number of sessions the participants had spent using the mobile application. As a result, 36 students used the application for 28 or more sessions, and only three students spent the time with the application less than required due to their illness on some days (number of sessions: Min=24, Max=67; Mean=31.81, SD=8.19; number of hours in total: Min=12, Max=37.17, Mean=17.36, SD=5.64).

After 4weeks, each student in the experimental and control groups was asked to complete the post-test, which was similar to the pre-test described above.

### Data Analysis

The Shapiro–Wilk test of normality and the Levene’s test of homogeneity of variance showed that the dataset was appropriate for parametric tests. After comparing the baseline with *t*-tests, we performed the mixed analysis of variances (ANOVAs) on the survey data and test scores to analyze and compare students’ psychological well-being (involving positive and negative affect) and cognitive performances (involving flow state, attention, and working memory) by the two conditions (i.e., experiment and control group) and two tests (i.e., pre- and post-tests). For the significant interaction effects, the Cohen’s *ƒ* effect size (Cohen, 1992) was calculated.

## Results

Descriptive statistics of the main outcome variables of the pre- and post-tests from both experimental and control groups were presented in Table 1. The results of the t-tests used to check baseline difference between the two groups reflected in the pre-test were also presented in Table 1. The *t*-test results showed that the randomization was achieved for most variables, e.g., positive affect [t(69)=0.36, *p*>0.05], negative affect [t(69)=0.10, p>0.05], orienting [t(69)=−0.25, p>0.05], conflict monitoring [t(69)=−1.04, p>0.05], and working memory accuracy [t(69)=−0.81, p>0.05]; however, there were differences between the two groups in alerting [t(69)=2.24, *p*<0.05] and working memory reaction time [t(69)=2.76, *p*<0.01]. Therefore, we decided to use mixed ANOVAs to further process the collected data, which took the baseline differences into consideration and focused on the interaction between group (i.e., experiment and control groups) and test time (i.e., pre- and post-tests).

**Table 1:** Descriptive statistics of pre- and post-test for both groups and *t*-test for pre-test.

Measures	Group	Pre-test		Post-test		Pre-test		
		Mean	SD	Mean	SD	*t*	*df*	*p*
Positive affect	Experimental	3.00	0.50	3.19	0.57	0.36	69	0.72
	Control	2.98	0.53	3.01	0.62			
Negative affect	Experimental	2.03	0.67	1.98	0.57	0.10	69	0.33
	Control	1.96	0.57	1.96	0.57			
Flow state	Experimental	3.14	0.62	3.31	0.45	−0.25	69	0.81
	Control	3.17	0.47	3.08	0.44			
Alerting (ms)	Experimental	27.08	20.36	19.42	19.00	2.24^*^	69	0.03
	Control	16.51	19.40	22.34	16.47			
Orienting (ms)	Experimental	18.00	23.42	18.10	22.73	−0.81	69	0.42
	Control	22.33	21.37	27.43	18.86			
Conflict monitoring (ms)	Experimental	62.52	24.19	61.21	20.12	−1.04	69	0.30
	Control	68.55	24.82	62.84	20.08			
Working memory accuracy (%)	Experimental	84.26	10.18	92.59	7.66	−1.17	69	0.25
	Control	86.79	7.94	90.10	8.90			
Working memory reaction time (ms)	Experimental	883.30	125.64	734.47	120.09	2.76^**^	69	0.01
	Control	783.75	175.05	714.06	109.04			

*SD=standard deviation*.

* *p<0.05*;

** *p<0.01*.

### Psychological Well-Being

**Figure 2:**
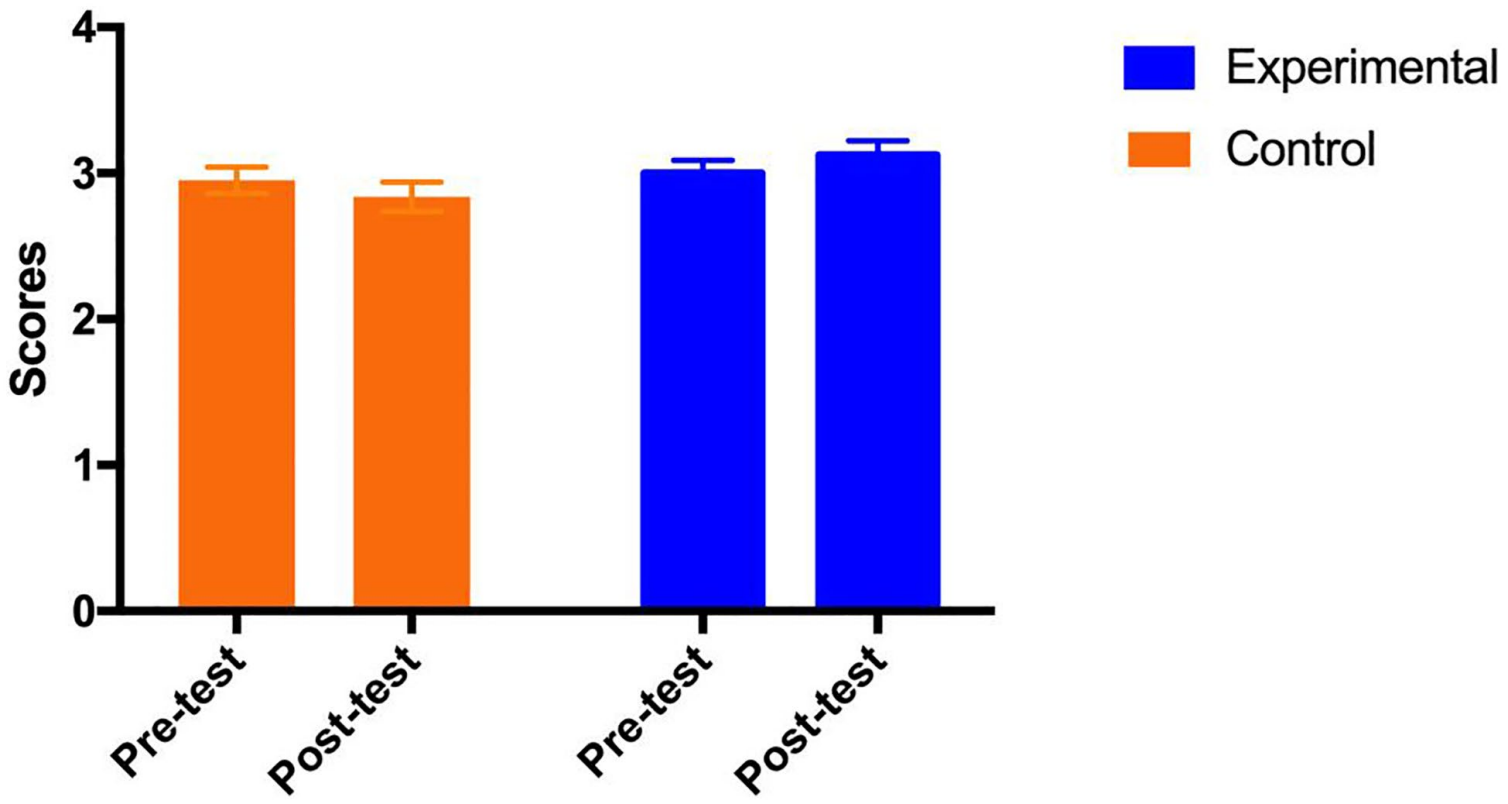
Adjusted means (SEs) for positive affect by group and test.

Mixed ANOVAs were conducted on both positive and negative affect scores separately. For the *positive affect*, the results show that there were no main effects of both group [*F* (1, 69)=2.69, *p*=0.11, η_p_^2^=0.038] and test time [F (1, 69)=0.38, *p*=0.54, η_p_^2^=0.005]. However, the interaction between group and test time was significant [*F* (1, 69)=6.86, *p*=0.01<0.05, η_p_^2^=0.090]. As shown in Figure 2, the *post hoc* analysis indicates that the positive affect in the control group remained stable [Adjusted Mean_pre_ (SE)=2.95 (0.09); Adjusted Mean_post_ (SE)=2.84 (0.10), *p*=0.16], while the positive affect in the experimental group was significantly improved from pre-test to post-test [Adjusted Mean_pre_ (SE)=3.00 (0.09); Adjusted Mean_post_ (SE)=3.12 (0.10), *p*=0.02] with a medium effect size (Cohen’s ƒ=0.31).

For the negative affect, the results show non-significant findings on neither the main effect of group [*F* (1, 69)=0.55, *p*=0.46, *η_p_*^2^=0.008] and test time [*F* (1, 69)=0.00, *p*=0.99, *η_p_*^2^=0.000], nor the interaction between group and test time [*F* (1, 69)=0.50, *p*=0.48, *η_p_*^2^=0.007].

### Cognitive Performance

#### Flow State

**Figure 3:**
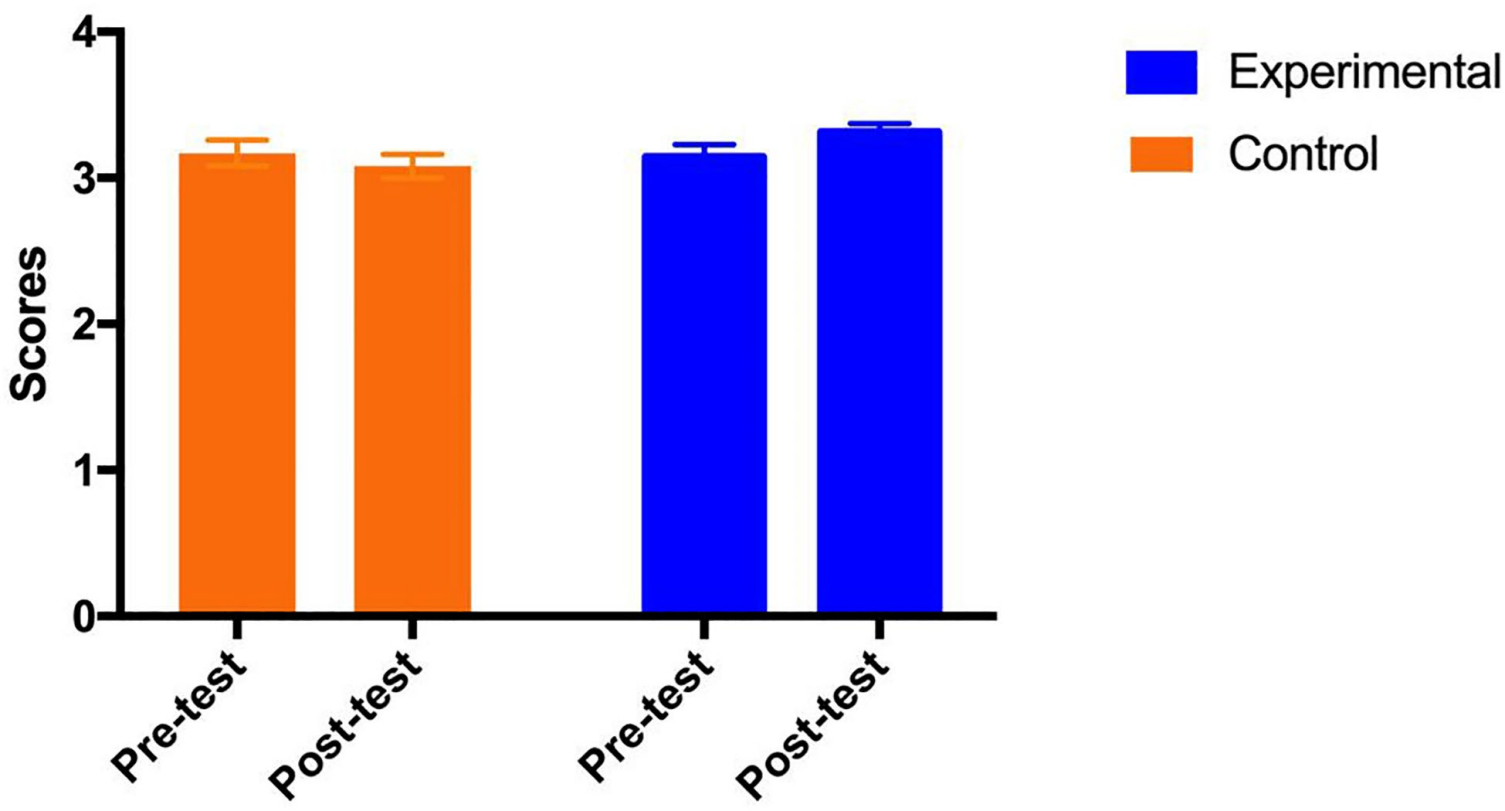
Adjusted mean (SE) for flow state by group and test.

Mixed ANOVA was performed on the flow state score. The results show that there were no main effects of both group [*F* (1, 69)=0.97, *p*=0.33, *η_p_*^2^=0.014] and test time [*F* (1, 69)=0.44, *p*=0.51, *η_p_*^2^=0.006]. However, the interaction between group and test time was significant [*F* (1, 69)=4.27, *p*=0.04<0.05, *η_p_*^2^=0.058]. As shown in Figure 3, the *post hoc* analysis indicates that the flow state in the control group remained stable [Adjusted Mean_pre_ (SE)=3.17 (0.09); Adjusted Mean_post_ (SE)=3.08 (0.08), *p*=0.33], while the flow state in the experimental group was marginally improved from pre-test to post-test [Adjusted Mean_pre_ (SE)=3.14 (0.09); Adjusted Mean_post_ (SE)=3.31 (0.07), *p*=0.06] with a medium effect size (Cohen’s *ƒ*=0.25).

#### Attention Performance

**Figure 4:**
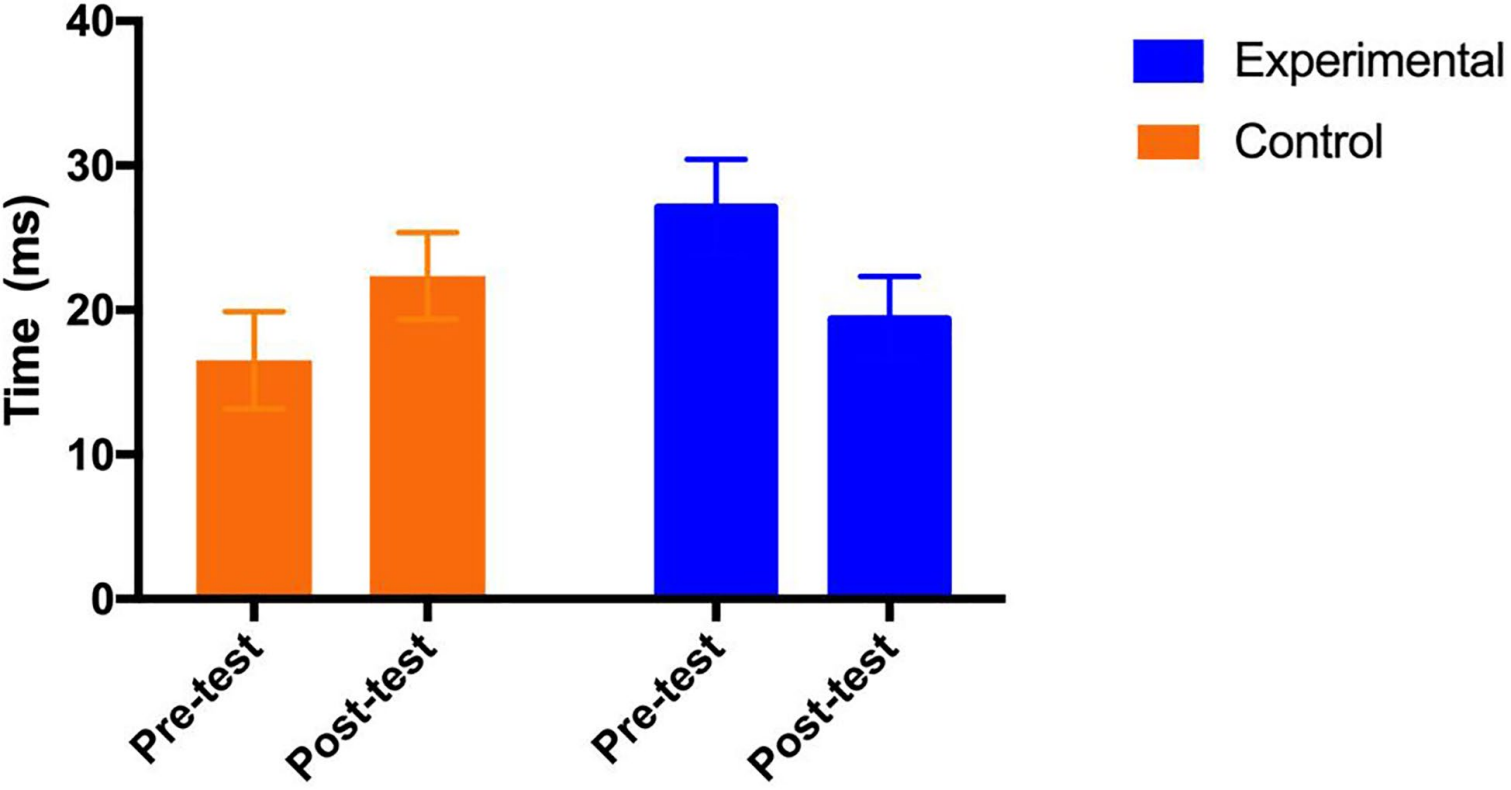
Adjusted means (SEs) for alerting by group and test.

Mixed ANOVAs were performed on the attention performance scores in terms of alerting, orienting and conflict monitoring. The results on alerting show that there was significant effect for the interaction between group and test time [*F* (1, 69)=7.06, *p*=0.01<0.05, *η_p_*^2^=0.093], and no main effects of both group [*F* (1, 69)=0.00, *p*=0.72, *η_p_*^2^=0.002] and test time [*F* (1, 69)=0.13, p=0.72, *η_p_*^2^=0.002]. As shown in Figure 4, the *post hoc* analysis show that the reaction time (for alerting score) of the control group had increased from pre-test and post-test, and the difference was approaching to but not quite statistically significant [Adjusted Mean_pre_ (SE)=16.51 (3.36); Adjusted Mean_post_ (SE)=22.34 (3.00), p=0.11]; on the contrary, the reaction time (for alerting score) of the experimental group significantly decreased from pre-test to post-test [Adjusted Mean_pre_ (SE)=27.08 (3.32); Adjusted Mean_post_ (SE)=19.34 (2.97), *p*=0.04] with a medium effect size (Cohen’s *ƒ*=0.32).

With respect to orienting, none of the effects were significant: main effect of group [*F* (1, 69)=2.79, *p*=0.10, *η_p_*^2^=0.039]; main effect of test time [*F* (1, 69)=0.69, *p*=0.41, *η_p_*^2^=0.010], and interaction between group and test time [*F* (1, 69)=0.64, *p*=0.43, *η_p_*^2^=0.009]. Similar results were obtained for conflict monitoring, i.e., none of the effects were significant: main effect of group [*F* (1, 69)=0.71, *p*=0.40, *η_p_*^2^=0.001]; main effect of test time [*F* (1, 69)=1.63, *p*=0.21, *η_p_*^2^=0.023], and interaction between group and test time [*F* (1, 69)=0.64, *p*=0.43, *η_p_*^2^=0.009].

#### Working Memory Performance

**Figure 5:**
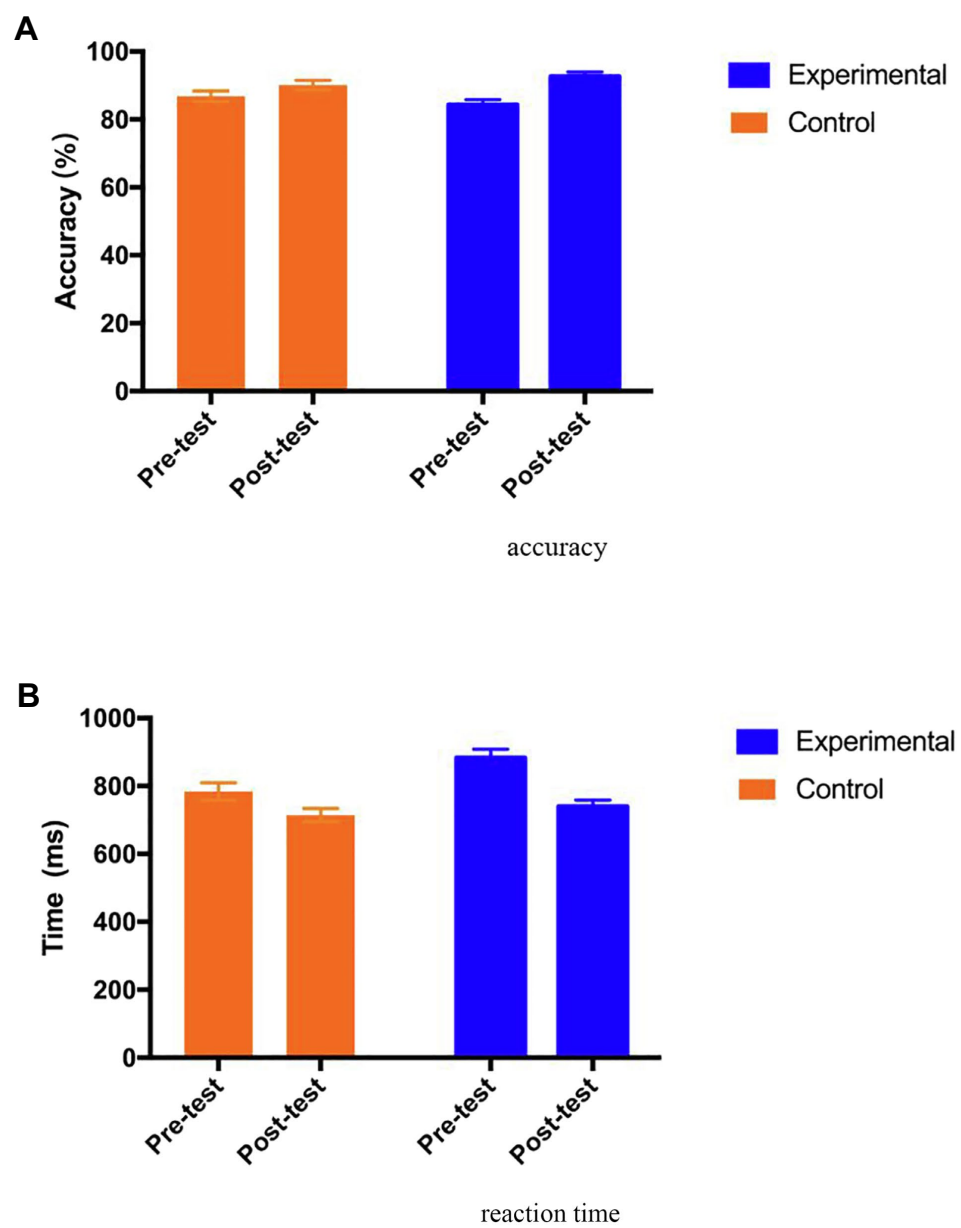
Adjusted means (SEs) for working memory accuracy **(A)** and reaction time **(B)** by group and test.

The working memory performance was assessed in terms of accuracy and reaction time. For the accuracy score, the Mixed ANOVA results show no main effect of group [*F* (1, 69)=0.00, *p*=0.99, *η_p_*^2^=0.000], but the effect of test time was significant [*F* (1, 69)=29.88, *p*=0.00, *η_p_*^2^=0.30]. Furthermore, there was significant interaction effect of group and test time [*F* (1, 69)=5.59, *p*=0.02<0.05, *η_p_*^2^=0.075]. As shown in Figure 5A, the *post hoc* analysis indicates that the accuracy score in the control group was improved significantly from pre-test [Adjusted Mean (SE)=86.79 (1.55)] to post-test [Adjusted Mean (SE)=90.10 (1.40); *p*=0.03<0.05], and the accuracy score in the experimental group was also improved significantly from pre-test [Adjusted Mean (SE)=84.26 (1.52)] to post-test [Adjusted Mean (SE)=92.59 (1.38); *p*=0.000<0.001], with a medium effects size (Cohen’s *ƒ*=0.28). While both groups improved the accuracy score, the experimental group improved significantly more than the control group.

With respect to reaction time, the results show that there were main effects of group [*F* (1, 69)=5.36, *p*=0.02, *η_p_*^2^=0.072] and test time [*F* (1, 69)=38.75, *p*=0.00, *η_p_*^2^=0.36]. Furthermore, the interaction effect of group and test time was significant [*F* (1, 69)=4.67, *p*=0.03<0.05, *η_p_*^2^=0.063]. As shown in Figure 5B, the *post hoc* analysis indicates that reaction time in the control group decreased from pre-test [Adjusted Mean (SE)=783.75 (25.69)] to post-test [Adjusted Mean (SE)=714.06 (19.40); *p*=0.006<0.01], and reaction time in the experimental group also decreased significantly from pre-test [Adjusted Mean (SE)=883.30 (25.34)] to post-test [Adjusted Mean (SE)=739.47 (19.13); *p*=0.000<0.001] with a medium effect size (Cohen’s *ƒ*=0.26).

## Discussion

The results of the study show that after the 4-week exposure to nature sounds through the mobile application, students in the experimental condition outperformed their counterparts in the control groups on psychological well-being and cognitive performance in some respects. The details of the findings under each of the research questions are discussed as follows.

**RQ1: Does exposure to nature sounds through a mobile application in daily life impact on psychological well-being among university students?**

The first research question examined the effects of using a nature-sound mobile application on university students’ psychological well-being in terms of both positive and negative affect. The results indicate that students who had used the nature-sound mobile application in daily life for 4weeks significantly improved their positive affect. The result is in accordance with SRT, which suggests that natural environments have restorative effects in physiological aspects (Ulrich, 1983; Ulrich et al., 1991). Similar findings were reported in previous studies on the effects of walking in the natural environment on positive affect (e.g., Hartig et al., 2003; Berman et al., 2008). Besides, Jo et al. (2019) reported that exposure to the sound of the forest could bring psychological benefits (e.g., relaxation and feelings of comfort), which have a link with positive affect.

The results show no difference in negative affect between the experimental and control groups. The negative affect reported by the participants in a five-point Likert scale was relatively low in both conditions (Mean=1.96, SD=0.58), with little space to explore significant effects. Further research may consider exploring the effects of nature-sound mobile applications on those people suffering from negative affect.

**RQ2: Does exposure to nature sounds through a mobile application in daily life impact on cognitive performance among university students?**
**RQ2.1: Does exposure to nature sounds through a mobile application in daily life increase flow state among university students?**

The results of the study indicate that students who had used the nature-sound mobile application in daily life for 4weeks significantly improved their flow state. The finding is consistent with Attention Restoration Theory that exposure to natural environments can help restore attention and improve cognitive performance. It also supports the finding of prior studies that exposure to nature would be helpful for people to reach a state of flow with quantitative evidence (Laumann et al., 2003; Schatz, 2019; Ruvimova et al., 2020). As reported by Ruvimova et al. (2020), presenting the nature scene (e.g., Hawaiian beach) in virtual reality helped workers to foster flow state in the open office settings. This study further suggested that the use of nature sounds would improve university students’ flow state while studying.

**RQ2.2: Does exposure to nature sounds through a mobile application in daily life improve students’ attention performance?**

The results of the study indicate that after using the nature-sound mobile application in daily life for 4weeks, the participants reduced their reaction time, showing the improved ability to achieve and maintain an alert state. However, there were no differences in orienting and conflict monitoring performances between the experimental and control groups.

According to the literature, there are two types of attention, i.e., voluntary and involuntary (James, 1892). They are distinguished in ART which suggests that voluntary attention is effortful and subject to fatigue, while involuntary attention is effortless and can facilitate the recovery of the attentional system (Kaplan and Kaplan, 1989; Kaplan, 1995). It is noted that both alerting and orienting require only involuntary attention (Emfield and Neider, 2014), and conflict monitoring requires directed or voluntary attention (Berto, 2005; Kaplan and Berman, 2010). However, the improvement in attention after exposure to nature elements has been mostly reported in terms of voluntary attention. For example, in a study using ANT to measure the benefits to attention after exposure to nature pictures, only conflict monitoring after viewing nature was observed while comparing to urban areas (Berman et al., 2008). According to a systematic review (Ohly et al., 2016), there are a variety of cognitive measures (e.g., digit-span and color Stroop) used for investigating the attention restoration potential of exposure to natural environments, and most of them targeted on executive function, which is highly related with voluntary attention (e.g., Taylor et al., 2002; Berman et al., 2008). However, this study did not find similar effects on conflict monitoring (a type of voluntary attention). With the similar ANT task, this result is in line with the finding of Emfield and Neider (2014), but inconsistent with the result of Berman et al. (2008). Since the work of Berman et al. (2008) has been done with a real walk in the park, this study, as well as the work of Emfield and Neider (2014), was conducted with simulated nature elements. Future studies should further explore whether there is a difference between real nature exposure and simulated experience on conflicting monitoring.

Furthermore, this study found that exposure to nature sounds improved alerting performance among university students after they had used a nature-sound mobile application for 4weeks. This result is not expected because according to the literature on ART, the improvement in attention after exposure to nature elements has been primarily reported in terms of voluntary attention, which often stems from the involuntary attention or fascination; thus, involuntary attention should not change (Kaplan and Kaplan, 1989; Kaplan, 1995). The unexpected result might be caused by the baseline difference in the alerting performance between the two groups. On the other hand, the literature on SRT has also suggested that exposure to nature environments can elicit high levels of involuntary attention (e.g., Ulrich et al., 1991). Our finding might be explained by the extended interventions (i.e., repeated experiences with nature sounds) that gave students a chance to exercise their involuntary attention, resulting in improvements that might not be observable with limited exposures to nature.

In terms of another type of involuntary attention, i.e., orienting, this study found no effects on this aspect which echoed the finding reported by Berman et al. (2008). These findings reveal the complexity of attentional benefits associated with the nature elements and measurement. It should also be noted that this study is among the very few studies that explore the use of nature sounds on university students in their daily life; and it lasts for 4weeks, which is longer than most of the existing studies. Future research is still needed on attention related issues.

**RQ2.3: Does exposure to nature sounds through a mobile application in daily life improve students’ working memory performance?**

The results indicate that students who had used the nature-sound mobile application in daily life for 4weeks significantly improved their working memory accuracy and reduced their reaction time. These results were highly aligned with both ART and the findings from many empirical studies. For example, previous studies have demonstrated the attention restoration effects of nature using various working memory tasks, e.g., digital-span and search and memory tasks etc. (Berman et al., 2008; Emfield and Neider, 2014; Abbott et al., 2016; Ohly et al., 2016; Van Hedger et al., 2019). The findings from this study also suggested an alternative option, i.e., a 2-back task, to measure the restorative effects on working memory for future studies.

## Conclusion

This study examined the effects of using a nature-sound mobile application in supporting university students’ psychological well-being and cognitive performance. The results show that after using the nature-sound mobile application in daily life for 4weeks, students in the experimental group outperformed their counterparts in the control group on psychological well-being reflected in positive affect, as well as cognitive performance reflected in flow state, attention (in terms of alerting), and working memory (in terms of accuracy and reaction time). The findings reveal the positive impact of exposure to relaxing nature sounds on university students’ psychological well-being and cognitive performance, as well as the potential of mobile applications to provide easy exposure to nature sounds.

The findings of the study have two implications. *First*, while the literature has reported restorative effects of exposure to the natural environment on mental fatigue, both visual elements (e.g., natural scene and pictures) and auditory elements (e.g., nature sounds) of the natural environment can be utilized to provide restorative experience. *Second*, with the wide use of information technology in particular mobile devices in recent years, mobile applications can be used to provide easy exposure to nature sounds, the auditory part of the nature environment in daily life, which has shown the potential to improve psychological being and cognitive performance among university students in this study. The findings of this study support the use of nature-sound mobile applications among university students to deal with the increasing challenges and to restore from mental fatigue in their daily lives.

Nevertheless, this study has some limitations. *First*, the participants were randomly assigned to two conditions without controlling their pre-study differences observed in cognitive tasks (i.e., alerting and working memory reaction time), in which the nature intervention might exert an influence. Such baseline differences may influence the result of the study. *Second*, the study did not examine how individual differences (i.e., personality, pre-experience, and intensity of usage) might influence the effects of using the nature-sound mobile application on university students’ psychological well-being and cognitive performance. Further studies should address these issues. *Third*, this study used questionnaires and cognitive tasks to examine the effects. Future studies should consider involving other measures such as physiological indicators (heart rate or skin-conductance levels) in the investigation. *Fourth*, the control group in this study did not receive any treatment, which might influence the result. The passive control may limit the findings in that the additional planning of daily routine required by an active task (e.g., playing a nature-sound mobile application during academic tasks) may influence the participants’ performance measured in this study. A future study will address this issue through the design of an active task for the control group. Finally, this study only used pre- and post-tests to examine the effects. Further studies will consider follow-up tests to examine whether there are any sustainable benefits.

## Data Availability Statement

The raw data supporting the conclusions of this article will be made available by the authors, without undue reservation.

## Ethics Statement

The studies involving human participants were reviewed and approved by the Ethics Review Committee of the Health Science Center, Shenzhen University. The patients/participants provided their written informed consent to participate in this study.

## Author Contributions

JL designed and conducted the study, collected and analyzed the data, and drafted the manuscript. MW conceptualized the research framework and revised the manuscript. LC contributed to the revision of the manuscript All authors contributed to the article and approved the submitted version.

## Funding

This research was supported by the Funding for Priority Topics in Beijing’s 14th Five-Year Plan for Educational Science (No. CHEA21017), National Natural Science Foundation of China (No. 61977023), and Eastern Scholar Chair Professorship Fund (No. JZ2017005) from the Shanghai Municipal Education Commission of China.

## Conflict of Interest

The authors declare that the research was conducted in the absence of any commercial or financial relationships that could be construed as a potential conflict of interest.

## Publisher’s Note

All claims expressed in this article are solely those of the authors and do not necessarily represent those of their affiliated organizations, or those of the publisher, the editors and the reviewers. Any product that may be evaluated in this article, or claim that may be made by its manufacturer, is not guaranteed or endorsed by the publisher.
